# Amino acid permeases in *Cryptococcus neoformans* are required for high temperature growth and virulence; and are regulated by Ras signaling

**DOI:** 10.1371/journal.pone.0211393

**Published:** 2019-01-25

**Authors:** Crislaine Lambiase Calvete, Kevin Felipe Martho, Gabrielle Felizardo, Alexandre Paes, João Miguel Nunes, Camila Oliveira Ferreira, Marcelo A. Vallim, Renata C. Pascon

**Affiliations:** 1 Universidade de São Paulo, Biotechnology Graduate Program, São Paulo, SP, Brazil; 2 Universidade Federal de São Paulo, Campus Diadema, Department of Biological Sciences, Diadema, SP, Brazil; Yonsei University, REPUBLIC OF KOREA

## Abstract

Cryptococcosis is an Invasive Fungal Infection (IFI) caused by *Cryptococcus neoformans*, mainly in immunocompromised patients. Therapeutic failure due to pathogen drug resistance, treatment inconstancy and few antifungal options is a problem. The study of amino acid biosynthesis and uptake represents an opportunity to explore possible development of novel antifungals. *C*. *neoformans* has 10 amino acids permeases, two of them (Aap3 and Aap7) not expressed at the conditions tested, and five were studied previously (Aap2, Aap4, Aap5, Mup1 and Mup3). Our previous results showed that Aap4 and Aap5 are major permeases with overlapping functions. The *aap4Δ/aap5Δ* double mutant fails to grow in amino acids as sole nitrogen source and is avirulent in animal model. Here, we deleted the remaining amino acid permeases (*AAP*1, *AAP*6, *AAP*8) that showed gene expression modulation by nutritional condition and created a double mutant (*aap*1Δ/*aap*2Δ). We studied the virulence attributes of these mutants and explored the regulatory mechanism behind amino acid uptake in *C*. *neoformans*. The *aap1*Δ*/aap2*Δ strain had reduced growth at 37°C in L-amino acids, reduced capsule production and was hypovirulent in the *Galleria mellonella* animal model. Our data, along with previous studies, (i) complement the analysis for all 10 amino acid permeases mutants, (ii) corroborate the idea that these transporters behave as global permeases, (iii) are required during heat and nutritional stress, and (iv) are important for virulence. Our study also indicates a new possible link between Ras1 signaling and amino acids uptake.

## Introduction

*Cryptococcus neoformans* is a cosmopolitan yeast with the ability to infect humans and animals. Cryptococcosis is the disease caused by this pathogen, which is a systemic infection, often fatal in immunodeficient population. The disease progression leads to invasion of the Central Nervous System, causing cryptococcal meningitis, which is the second main cause of death after tuberculosis [1–5]. In order to invade the host, *C*. *neoformans* expresses several phenotypic features that guarantee successful colonization. In this context, metabolic versatility is an important feature, allowing the pathogen to use several carbon and nitrogen sources, as well as other nutritional elements, such as iron, phosphate, sulfur, amino acids etc., which are important to survival, but they may not be abundant in the animal host [6,7]. Also, the production of a polysaccharide capsule is required to overcome the immune system, and help the yeast to escape phagocytosis by the alveolar macrophages [8]. The ability to cope with stress factors is also an essential characteristic for survival, such as resistance to high temperature, oxidative and osmotic stress [9]. Other virulence factors are considered important, such as, production of urease and phospholipase [10,11]. Failure to express these traits results in avirulence or hypovirulence in animal model as *G*. *mellonella* and murine [12–19]. The ability to sporulate through the sexual cycle and haploid fruiting is also very important since the production of spores is linked to the dissemination of *C*. *neoformans* and its inhalation is the most common form of contamination [20].

Once the disease is established in the host, the treatment of IFIs, especially Cryptococcosis, is difficult due to the high toxicity of the antifungals and high rate of acquired resistance [19,21,22]. This situation requires the search for novel antifungal drugs that are effective at low dosages and have fewer side effects [19,21,23]. Amino acid uptake and biosynthesis pathways as molecular targets for antifungal development have been explored in the last decade due to the absence of some of these biosynthetic routes in animal cells [24–32]. Among others, the tryptophan and sulfur amino acids biosynthesis pathways have been shown as useful targets, since interruptions of these biosynthetic routes are either essential (tryptophan) or render strains avirulent (methionine) in animal model. Also these biosynthetic pathways are absent in higher eukaryotes, suggesting that drugs acting upon these biochemical process will have high selective toxicity [29–31]. In fact, our previous work has shown that the use of tryptophan biosynthesis inhibitors can promote growth arrest in *C*. *neoformans* at low concentrations [31].

Along these lines, we have also studied the amino acid uptake system that operates in *C*. *neoformans*. Our group has found 10 amino acid permeases genes that encode transporters belonging to the APC (Amino Acid-Polyamine-Choline) family [31,33,34]. Expression analysis have shown that 2 of them (Aap3 and Aap7) have no expression during nutritional stress. However, Do et al. (2016) found that Aap3 is induced in the presence of lysine, a condition that we have not tested [35]. Aap4 and Aap5 have 89% of amino acids similarity, overlapping function and are required for amino acid uptake at high temperature (37°C). A strain containing a double deletion of these genes is sensitive to oxidative stress, high temperature and is avirulent in *G*. *mellonella* and murine animal model [33].

In *S*. *cerevisiae*, Gap1 is the main global permease, which acts with low specificity and broad range transport of amino acid [36–38]. Gap1 also functions as a transceptor, signaling to the PKA pathway [39,40]. At this point in *C*. *neoformans*, the permeases that have been studied so far seem to function as Gap1, that is, as global permeases.

The regulatory mechanism and the genetic elements that are activated and trigger amino acid uptake have not been broadly studied in *C*. *neoformans*. What is known so far is that these permeases are transcriptionally induced during nitrogen starvation, removal of nitrogen catabolite repression (ammonium sulfate) or in the presence of the substrate (amino acids) [31]. In *S*. *cerevisiae*, these same cues are known to activate signaling systems that lead to several physiological changes, among which, is the induction of amino acid uptake by permeases. For example, nitrogen deprivation activates Global Amino Acid Control (GAAC), where the main player is the Gcn4 transcription factor [41]; the presence of preferred nitrogen source (ammonium sulfate) leads to nitrogen catabolite repression (NCR) of genes related to the acquisition of alternative nitrogen sources, such as amino acids by the Gata1 transcriptional factor [37,41,42]. This system is also known in *C*. *neoformans* [43] and likely play a role on permease gene expression; however, that remains to be shown.

In *S*. *cerevisiae*, the presence of amino acids in the environment also activates the SPS-sensing mechanism, a signaling system that causes permease gene expression, favoring amino acid uptake. This system is composed of a transceptor (Ssy1), a protease (Ptr5) and two Zinc-finger transcription factors (Stp1 and Stp2) [42]. In *C*. *albicans* there are 6 GAP1-like permeases, however only GAP2 is considered to be a *S*. *cerevisiae* Gap1 homologue. The knockout of GAP2 leads to amino acid transport failure and morphogenetic abnormalities. In this opportunistic pathogen, GAP2 is regulated by NCR and a SPS-sensing system similar to *S*. *cerevisiae* [44,45].

In *C*. *neoformans*, amino acid uptake is important for growth during carbon and nitrogen stress. Thermotolerance and capsule synthesis are also dependent on amino acid uptake, which highly contribute to virulence in animal models [33]. The functionality of the APC-like transporters in *C*. *neoformans*, whether global or specific amino acid transporters, remains to be shown, since not all nutritional regulated permeases have been studied.

In order to have a complete set of data on amino acid permeases in *C*. *neoformans*, we characterized the nature and relevance of *AAP*1, *AAP*6, *AAP*8 for survival and virulence. Due to the amino acid similarity of the permease Aap1 and Aap2 (80.9%), we constructed a double mutant to test for overlapping functions. Our results confirmed that Aap1 and Aap2 are significantly required for amino acid uptake as sole nitrogen source at higher temperature; double deletions strains have reduced capsule and are hypovirulent in animal model.

Taken together, the phenotypic characterization of amino acid permeases in *C*. *neoformans* conducted in this work and previous reports [31,33,35], has shown that these transporters are mostly global and often redundant. They are highly regulated by the nutritional condition (carbon and nitrogen sources) and are important for virulence *in vitro* and *in vivo*. However, very little information is available in the literature concerning the regulation of amino acid uptake, in spite of its relevance for growth at 37°C and virulence. We tested two hypotheses to investigate the genetic regulation behind this process: (i) Stp1 and Stp2-like zinc-finger transcription factors found by homology-based and bioinformatics approach in *C*. *neoformans* genome play a role in amino acid uptake, and (ii) a cross talk exist between the regulatory mechanisms that governs thermotolerance and amino acid uptake in *C*. *neoformans*. Ras signaling controls a number of cell features important for morphogenesis and adaptation to the host, among them, high temperature growth is central for virulence [46–53]. By studying a *ras1*Δ mutant we were able to find novel nutritional phenotypes associated to Ras signaling, which have not been documented before. In this paper, we have started to uncover the regulatory mechanism of amino acid uptake, which could be interesting not only regarding the basic knowledge of the subject, since it is unexplored, but also for applied purpose. The identification of a regulatory mechanism could expand the possibilities of using this biological aspect as a drug target. In fact, conserved sequences among fungal Ras1 were recently identified and could be the target for inhibitor development, which would act upon the Ras1 protein of the pathogen but not the host, increasing selective toxicity [54]. In this paper we have completed the characterization of amino acid permeases and found a novel feature of Ras1 that will open an interesting avenue to explore the connection between nutrition, thermotolerance and Ras signaling.

## Materials and methods

### Strains, growth conditions, and genetic manipulations

All strains used and constructed in this work are *C*. *neoformans* var. *grubbii* serotype A (H99). They are listed in S1 Table. The *ras1*Δ::Neo^R^ (CNB045) strain was generously donated by Dr. Andrew Alspaugh [46]. Routine growth was carried out on rich medium YPD (1% yeast extract, 2% bacto-peptone, 2% glucose). Synthetic dextrose (SD) was prepared with yeast nitrogen base, YNB (0.67g/L yeast nitrogen base w/o amino acid and ammonium sulfate, 2% glucose, 10mM nitrogen source) and SG (synthetic galactose), dextrose was substituted by galactose. Unless specified, growth temperature used was 30°C or 37°C with 150 rpm in a rotary shaker. Spot dilutions were made by growing overnight cultures in YPD, cells were washed twice in sterile saline, adjusted to 2 x 10^6^ CFU/mL and serial diluted until to 2 x 10^2^ CFU/mL. Five microliters of each dilution were spotted on test plates.

Deletion constructs were generated by double-joint PCR as described before [55]. All primers used to generate the construct for gene deletion are listed in S2 Table. In brief, the coding sequence of the target gene (from start to stop codons) was substituted by a selectable marker (resistance to geneticin or hygromycin, 200 μg/mL) which was PCR amplified from plasmids pZPNeo or pZPHyg [56]. Homologous recombination regions (600 bp) corresponded to promoter and terminator sequences of the target genes were PCR amplified from H99 gDNA. Constructs were introduced in H99 strain by biolistic transformation according to previously reported protocol [57], selected in YEPD added with 1 M sorbitol and the appropriate antibiotics (Geneticin or hygromycin) depending on the selectable gene marker used for each gene deletion.

### Growth rate on amino acids

Single amino acid assimilation protocol was previously described [33]. In brief: the ability to grow on amino acid as sole nitrogen source was evaluated on 96 well plates in 100 μL total volume of SD or SG (2% dextrose or 2% galactose, respectively) and 10 mM of a single amino acid as sole nitrogen source, fifteen amino acids were tested, since five of them do not support growth (alanine, threonine, histidine, tyrosine and cysteine). Cells were grown overnight in YEPD at 30°C, washed 3 times in sterile PBS. Intracellular nitrogen pools were exhausted by incubation of the washed cells in PBS (Phosphate Buffered Saline) at 30°C with 150 rpm rotation for 2 hours. After this period, a total of 2x10^2^ cells were inoculated in each well containing a single amino acid as sole nitrogen source and a variable carbon source. All experiments were done in technical triplicates. Plates were incubated at 30 and 37°C for 48 hours. The OD_600_ was measured in a plate reader (Logen). A minimum of three biological replicates were done for all experiments. Assay controls: inoculums were cultivated on SD medium with ammonium sulfate for positive control and without any nitrogen source for the negative control in the same condition described above. All statistical analysis were performed using Two-way ANOVA multiple comparison test by GraphPad Prism 7.0 software and were considered statistically significant *p* values lower than 0.05.

### *In vitro* virulence and stress resistance assays

In order to evaluate capsule production, the cells were cultivated in YPD medium at 30°C with orbital shaking (150 rpm) overnight, they were collected by centrifugation, washed three times with PBS 1X and normalized to an OD_600_ of 0.3 in 1X CO_2_ independent medium (Gibco BRL) and incubated at 30°C and 37°C for up to 72 hours [58]. Cells were stained with BactiDrop India Ink (Remel) for capsule observation under the light microscope. Capsules were documented at 24, 48 and 72 hours using MIPro Standard v1.1 Software. Quantitative analysis of capsule diameter was performed as described before [59]. Urease, phospholipase and melanin production were evaluated according the published protocol [60]. Osmotic stress was evaluated on YPD plates supplemented with KCl or NaCl (0.75 and 1 M) and alkaline stress by raising the pH to 6, 7 and 8. Also, cell wall and plasma membrane integrity was evaluated in rich and synthetic medium supplemented with Congo red (0.5%) and SDS (0.03%), respectively. Oxidative stress was evaluated on YEPD and SD (added with ammonium sulfate only and ammonium sulfate plus amino acids) plates supplemented with hydrogen peroxide (1 and 2.5 mM). All experiments were done at 30 and 37°C in triplicates.

### *In vivo* virulence assay

*G*. *mellonella* was used for in vivo virulence experiments according to published protocol [61]. In brief, strains were inoculated into 5 mL of YEPD and incubated with orbital agitation 150 rpm for 16-18h. Cells were collected by centrifugation, washed twice in sterile PBS and adjusted to 1x10^6^cell/mL in PBS supplemented with ampicillin (20mg/kg body weight). Groups of 16 caterpillars with 200mg on average body weight were inoculated with 10μL of the inoculums with the aid of a Hamilton syringe in the region of the last pro-paw. Thereafter, caterpillars were separated on glass Petri dishes (15mm diameter) and incubated at 30°C and 37°C during 8 days. Caterpillars were monitored daily by observing spontaneous or provoked movements with the aid of a previously sterilized clip. The experiment was completed when the larvae die or formed cocoons.

### Mating and haploid fruiting

Mating and the haploid fruiting experiments were conducted in accordance with previous reports [62]. Mutant strains (mating type alpha) were mated to KN99a, control mating experiments were done with H99, KN99α and KN99a. In brief, strains were inoculated into 5 mL YEPD and incubated with orbital agitation 150 rpm for 16-18h. The co-cultivation was done by joining 5 μL of a cell suspension (2 x10^6^ cells/mL) of each opposite mating type strains (mutant α + KN99a), the mixture was spotted on filament agar culture medium [63]. The plates were incubated at room temperature in the dark for 43 days and observed in a stereoscope (Optimus TM-30) and in a light microscope (Olympus BX51) 400 x and 1000 x magnification. For the haploid filamentation evaluation was made in the same condition described above using only one strains in the same concentration.

### Bioinformatics

Bioinformatics analysis was done at NCBI (National Center for Biotechnology Information) through the online platform Blastp. Protein domains and signature were identified at MotifScan online database (https://myhits.isb-sib.ch/cgi-bin/motif_scan), TMHMM Server v. 2.0 (http://www.cbs.dtu.dk/services/TMHMM-2.0/) and InterPro (https://www.ebi.ac.uk/interpro/). Amino acid sequence alignments were done with LaserGene software (DNAStar, Inc.)

### RNA extraction, cDNA synthesis and qPCR

Expression analysis was performed from total RNA extracted as follow: strains were incubated in liquid YEPD overnight in 150 rpm agitation. The cells were washed twice in sterile ultrapure water after centrifugation, and resuspended in the medium, which was adjusted to the appropriate temperature (30 or 37°C). Previously published protocol [64] for RNA extraction was followed with a few modifications: 3x10^8^ cells were grown for 2 hours in various medium conditions, centrifuged and resuspended in 750 μL Trizol (Invitrogen) and the same volume of glass beads. The mixture was homogenized ten times in vortex for 1 minute intercalated for 2 minutes on ice. The glass beads were precipitate at room temperature by gravity. A second Trizol extraction was done as before. The RNA was dissolved in DEPC-treated water and stored at—20°C for short term use and at– 80°C for long storage. RevertAid H minus First Strand cDNA synthesis kit was used for cDNA synthesis (Thermo Scientific), Oligo dT, random hexamer primers and 5 μg of total RNA. Real time PCR amplifications were made from diluted templates (1:10) with 600 nM target primers, 300 nM *GPDH*1 (Glyceraldehyde-3-phosphate dehydrogenase) internal control primers, and 1X HOT FIREPol EvaGreen qPCR Mix Plus ROX (Solis Biodyne—08-24-00001). The quantification was performed by the ΔΔ^CT^ method, normalization as done against *GPDH*1, as previously described [65]. One-way ANOVA multiple comparison tests using GraphPad Prism 7.0 software were used for analysis of variance and *p* values lower than 0.05 were considered statistically significant.

## Results

### *C*. *neoformans* amino acid permeases belong to the APC family

Our group reported earlier that amino acid permeases genes encoded by *C*. *neoformans* genome have a different organization compared to *S*. *cerevisiae* [31]. The later has 24 genes encoding permeases of the APC super family, which are not highly specific permeases. They transport a broad spectrum of amino acids or groups of biochemically related amino acids and D-isomers [38]. In addition, *S*. *cerevisiae* and *C*. *albicans* have Gap1, which is considered the global amino acid permease [39,41,45]. *C*. *neoformans* genome encodes 10 plasma membrane amino acid transporters of the APC family plus 3 genes that seems to encode GABA permeases (CNAG_01535, CNAG_02455 and CNAG_05017), which were found by BLASTp searches using the 24 *S*. *cerevisiae* sequences as query [31]. Also, various putative vacuolar permeases were found.

In this work, the same *C*. *neoformans* amino acid sequences were used to search the human genome at NCBI, and five genes encoding permeases of the APC superfamily were retrieved and presented various degrees of similarity to the *C*. *neoformans* permeases (Table 1). The most significant sequence similarity found with *H*. *sapiens* genes refers to Mup1 and two human permeases NP_055085.1 (36%) and NP_003974.3 (35%), followed by Aap1 and NP_066000.2, which in spite of slightly higher identity percentage (38%) compared to Mup1, presented a much lower query cover and E-values (Table 1). *C*. *neoformans* Mup1 and Mup3 permeases are considered homologues of *S*. *cerevisiae* cysteine and methionine permeases [66,67]. In *C*. *neoformans* Mup1 and Mup3 share 16.5% identity; they were considered global permeases, since a double mutant *mup*1Δ/*mup*3Δ showed growth deficiency in several amino acids as sole nitrogen sources including methionine [33].

**Table 1:** Summary of the amino acid sequence similarity among APC-like permeases from *C*. *neoformans* and *H*. *sapiens*.

*C*. *neoformans* ID	Description	Max score	Total score	Query cover	E value	Ident	*H*. *sapiens*
AAP1 (this work)	probable cationic amino acid transporter [*Homo sapiens*]	47.0	47.0	15%	3e-04	38%	NP_066000.2
AAP2 [33]	probable cationic amino acid transporter [*Homo sapiens*	46.6	46.6	61%	4e-04	25%	NP_066000.2
AAP3 [68]	Y+L amino acid transporter 2 [*Homo sapiens*]	32.7	32.7	42%	6.7	22%	NP_003974.3
AAP4 [33]	Y+L amino acid transporter 2 [*Homo sapiens*]	59.7	59.7	60%	2e-08	24%	NP_003974.3
AAP5 [33]	Y+L amino acid transporter 2 [*Homo sapiens*]	59.7	59.7	66%	2e-08	24%	NP_003974.3
AAP6 (this work)	solute carrier family 7 member 13 [*Homo sapiens*]	47.4	47.4	29%	2e-04	24%	NP_620172.2
AAP7 (not analyzed)	No hits found	-	-	-	-	-	-
AAP8 (this work)	Amino acid/metabolite permease [*Homo sapiens*]	70.5	70.5	50%	2e-11	24%	SJM32844.1
MUP1 [33]	B(0,+)-type amino acid transporter 1 [*Homo sapiens*]	264	264	75%	1e-80	36%	NP_055085.1
	Y+L amino acid transporter 2 [*Homo sapiens*]	263	263	76%	5e-80	35%	NP_003974.3
MUP3 [33]	Y+L amino acid transporter 2 [*Homo sapiens*]	102	102	79%	6e-22	27%	NP_003974.3
	B(0,+)-type amino acid transporter 1 [*Homo sapiens*]	95.5	95.5	75%	9e-20	26%	NP_055085.1

The other eight *C*. *neoformans* permeases (Aap1-8) have limited sequence similarity to human permeases as showed in Table 1. A phylogenetic tree (S1 Fig) depicting all related permeases in *C*. *neoformans* and *H*. *sapiens* shows that Aap1, 2, 3, 4 and 5 have higher percentage identity and are closer among themselves than the other permeases. Aap6 and Aap7 are in the same branch with 41.4% identity. AAP8 is clustered with two human permeases: SJM32844 and NP_066000.2 (19.9% and 17.2% identity, respectively) (S1 Fig). Therefore, the majority of *C*. *neoformans* and *H*. *sapiens* permeases have low identity percentages and low query cover in general, suggesting that these permeases might be sufficiently different to be considered as selective drug targets. [33].

**Fig 1:**
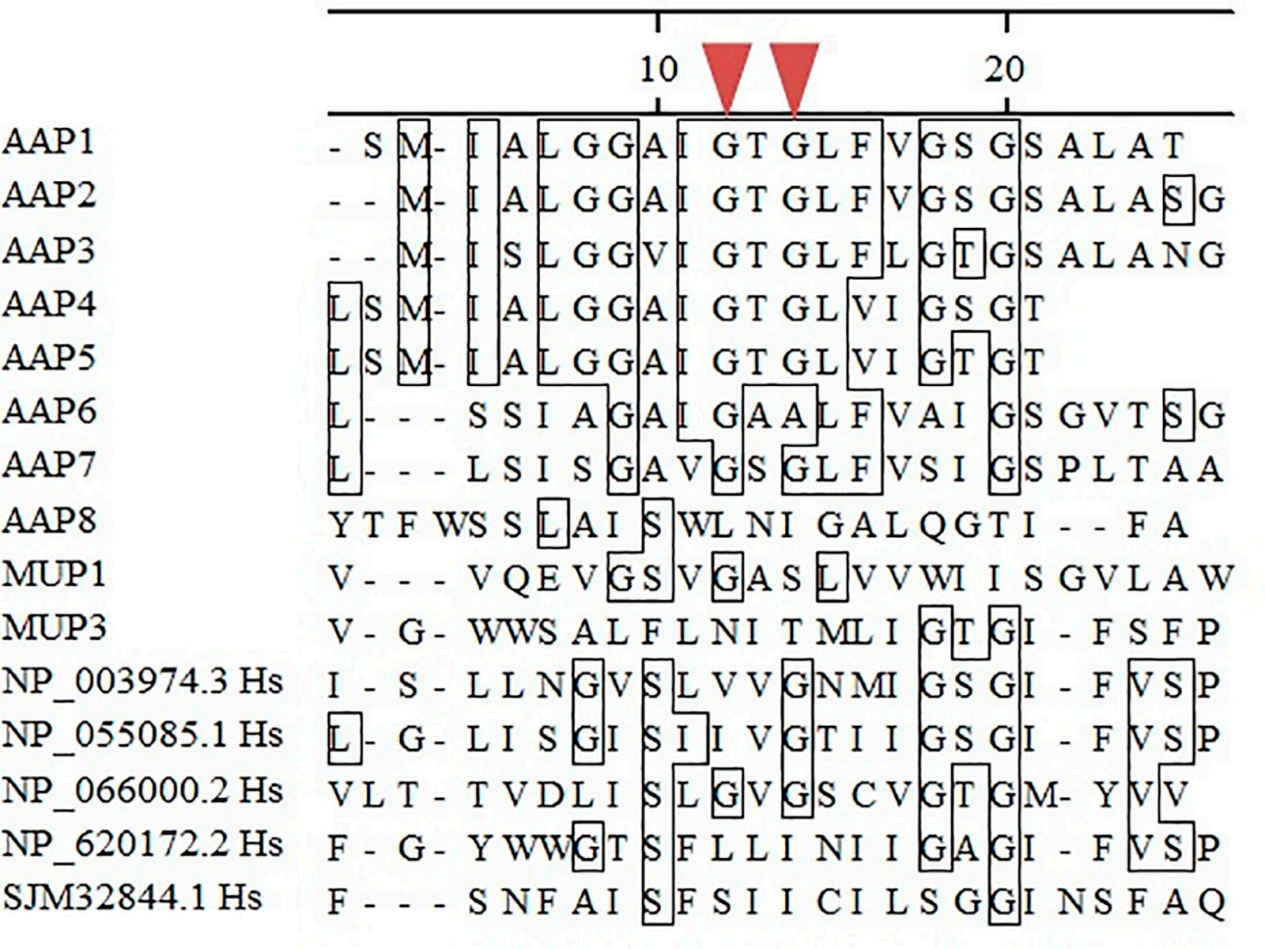
Amino acid sequence alignment. Conserved residues at transmembrane one (TM1) of *C*. *neoformans* and *H*. *sapiens* permeases of the APC family. The red inverted triangles represent glycine residues that are known to be invariable and essential for amino acid affinity and binding in *S*. *cerevisiae* APC permeases.

Gournas et al. (2018) reported that a limited number of amino acid residues located at five transmembrane segments of yeast APC transporters is required for amino acid binding and specificity [69]. The most relevant residues are at transmembrane segment 1 (TM1), with two glycines, which are invariable in 14 aligned sequences of the yeast APC superfamily. Fig 1 shows the amino acid sequence alignment of TM 1 for all 10 *C*. *neoformans* permeases and their counter parts in humans. The red triangles in Fig 1 show that these two residues are conserved for Aap1, Aap2, Aap3, Aap4, Aap5 and Aap7 but not for the remaining *C*. *neoformans* permeases. The glycine residues in *H*. *sapiens* are only conserved in NP_066000.2. This information, in association with the identity data presented in Table 1, suggests that *C*. *neoformans* and *H*. *sapiens* permeases are not only divergent regarding amino acid primary sequence, but are also different regarding conserved amino acid residues that are known to be functional, supporting the idea that the amino acid transporters could be molecular drug targets with high selective toxicity, which are useful for antifungal development.

### Deletion and growth phenotypes of *aap*1Δ, *aap*6Δ, *aap*8Δ and *aap*1Δ/*aap*2Δ

The knockout mutants used in this work were constructed by removing the coding region of Aap1, Aap6 and Aap8, individually, and also, a double mutant *aap*1Δ/*aap*2Δ was created due to high amino acid sequence similarity between these two genes. S2 Fig shows the Southern blots of each mutant constructed.

Growth of the single and double mutants (*aap1*Δ, *aap2*Δ, *aap6*Δ, *aap8*Δ and *aap1*Δ*/aap2*Δ) on solid rich medium and on synthetic dextrose (SD), either on preferred nitrogen source (ammonium sulfate) or on amino acids as sole nitrogen source showed no difference relative to the wild type after 48 hours of at 30°C and 37°C, indicating that these permeases are not essential for growth in the temperature and nutritional conditions tested.

Liquid cultures were used to determine the growth rate of the mutants in media containing 10 mM of each amino acid as the sole nitrogen source at 30°C and 37°C, compared to the wild type H99. Regarding *aap6*Δ and *aap8*Δ, no significant differences in growth were observed for all amino acids analyzed at 30°C. Whereas, growth in methionine and proline medium was decreased for the *aap*1Δ mutant, when two independent transformants (CNU103 and CNU108) were tested (methionine 41.26% and 36.90% and proline 77.55 and 77.44% respectively). This result suggests that some of these permeases might be required but not essential for growth on amino acids. One explanation for this result is that the presence of the other global permeases, which are efficient amino acid transporters, probably supports growth even when one of the permeases is missing. However, for two independent transformants (CNU114 and CNU116) of *aap*1Δ/*aap*2Δ there was a statistical significant growth reduction when glutamine (64% and 65% respectively) and arginine (42% and 40%, respectively) were used as independent sole nitrogen sources at 30°C (*p* < 0.05) compared to wild type.

Regarding growth at 37°C, there was no significant difference relative to wild type and *aap*6Δ; however, *aap*1Δ had growth differences compared to wild type in methionine media for both transformants CNU103 and CNU108 (67,17% and 62,17%, respectively). The single mutant *aap*8Δ had significant growth differences in methionine (66%), glutamic acid (71%) and tryptophane media (45%) (*p* < 0.001). As for the double mutant, *aap*1Δ/*aap*2Δ, there was a growth reduction in almost all amino acid media groups (9 out of 15), as shown in Table 2, indicating that the two permeases are essential for the growth on amino acids as sole nitrogen source at 37°C. These results suggest that Aap1 and Aap2 may have a compensatory effect, since the individual deletion of these permeases did not show the same pattern of growth reductions at 37°C as did the double mutant.

**Table 2:** Growth of two independent strains containing a double deletion (*aap*1Δ/*aap*2Δ) on single amino acid as sole nitrogen source.

Group	Amino acid	% of growth			
		CNU114		CNU116	
Non polar (hydrophobic)	Ala	-	-	-	-
	Gly	61	****	66	***
	Leu	68	**	60	****
	Ile	69	**	71	**
	Pro	90	NS	92	NS
	Val	36	****	35	****
Polar (Uncharged)	Asn	85	NS	86	NS
	Gln	58	****	55	****
	Ser	24	****	34	****
	Thr	-	-	-	-
Acid	Asp	68	**	71	**
	Glu	102	NS	92	NS
Basic	Arg	74	*	75	*
	His	-	-	-	-
	Lys	107	NS	106	NS
Sulfur	Met	88	NS	99	NS
	Cys	-	-	-	-
Aromatic	Phe	42	****	38	****
	Trp	119	NS	115	NS
	Tyr	-	-	-	-

(-) = not tested, NS = no statistical significant different relative to wild type and stars represent the statistical significance at * *p* < 0,03, ** *p* < 0,002, *** *p* < 0,0002 and **** *p* < 0,0001.

These observations are largely in agreement with our previous data, where we found that amino acid permeases are specially required at 37°C under growth in amino acids as sole nitrogen source. The deletion of two sets of permease genes that have high sequence similarities (*aap*4Δ/*aap*5Δ and *mup*1Δ/*mup*3Δ) caused growth reduction, especially at higher temperature [33]. The same was detected for the double mutant *aap*1Δ/*aap*2Δ constructed in this work, suggesting that amino acid permeases have overlapping functions and are important for amino acid uptake at 37°C. Also, our previous data indicates that temperature is an important factor for permease gene regulation, since growth at 37°C induces permease expression in synthetic medium [33].

### Permease phenotypes related to virulence and stress traits

We evaluated the ability of the permease mutant strains (*aap*1Δ, *aap*6Δ, *aap*8Δ and double mutant *aap*1Δ/*aap*2Δ) to express the key virulence factors that guarantee survival in the host. Regarding melanin, phospholipase B and urease production, we found no difference between mutants and wild type. Therefore, we concluded that these mutations do not affect the virulence factors analyzed so far.

The same mutant strains were tested for several stress factors which are present during host invasion and are important for pathogenesis [18]. The strains were inoculated on agar plates containing YEPD and/or variations of YNB added with stressing agents in order to simulate: oxidative stress (1 and 2.5 mM H_2_O_2_), alkaline stress (pH's 6, 7 and 8), saline and osmotic stress (0.75 and 1 M NaCl and KCl, respectively) and cell wall stress (SDS and Congo Red) at 30 and 37°C for 48 h. The study established that the absence of these permeases did not affect the *C*. *neoformans* growth in the presence of these various stressing agents.

All mutants were tested for the ability to enter the sexual cycle, an important feature in order to produce spores, which are infectious particles [20,70,71]. Also, the filament formation, important for mating and haploid fruiting, is induced by nitrogen starvation; therefore, we tested the mutants’ ability to mate to KN99a. All strains were able to mate and filament as wild type, demonstrating that these permeases are not required neither for filament production and nor for mating.

**Fig 2:**
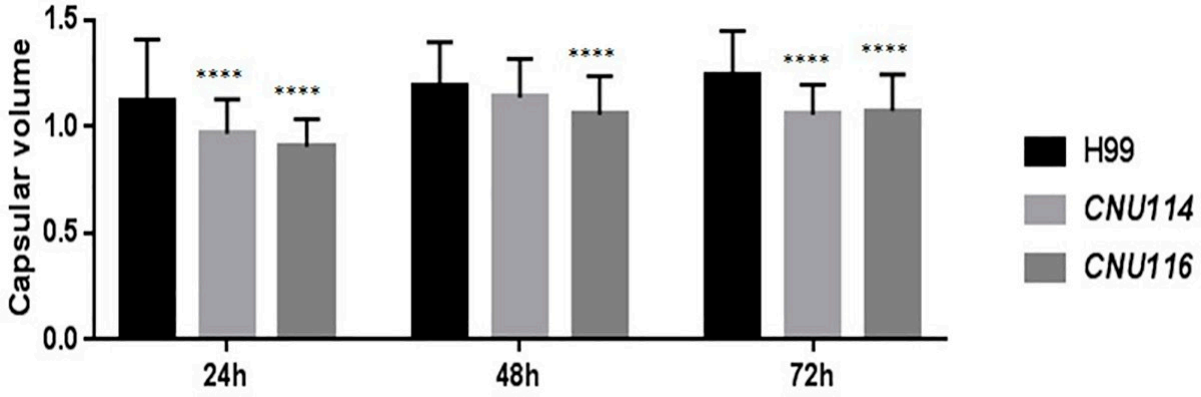
Capsule volume in wild type H99 and two independent transformants (CNU114 and CNU116) of a double mutant *aap*1Δ*/aap*2Δ at 37°C during 24, 48 and 72 hours. *p* < 0,001.

The polysaccharide capsule is considered a very important virulence factor in *C*. *neoformans* [8,72–74] since it provides protection to the pathogen by avoiding the host immune system. The capsule size was evaluated for all mutants tested in this work at 24, 48 and 72 hours in CO_2_-independent culture medium at 30 and 37°C. The results showed that only the double mutant *aap1*Δ*/aap2*Δ presented a reduction in capsular size at 37°C at all time points tested, compared to wild type H99 (Fig 2). This is consistent with our previous report in which two double mutant strains, *aap*4Δ/*aap*5Δ and *mup*1Δ/*mup*3Δ, were deficient in capsule production [33].

**Fig 3:**
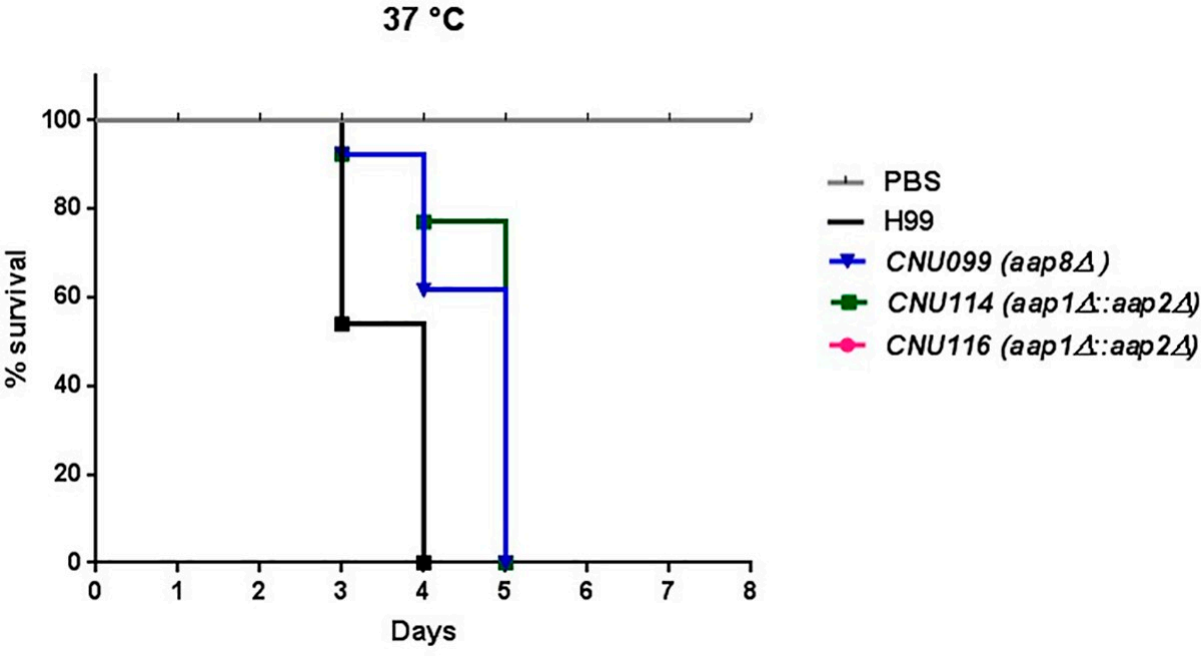
Animal model of infection in *G*. *mellonella*. The larvae were inoculated with the wild type (H99), single mutants (*aap6*Δ and *aap8*Δ) or double deletion (*aap1*Δ*/aap2*Δ) at 37°C for 8 days. Survival was followed during the course of the infection. Mutants *aap8*Δ and *aap1*Δ*/aap2*Δ were hypovirulent when compared to wild type H99 (**** and ***, respectively). PBS is a negative control.

This result led us to verify the virulence of the mutants in animal model *G*. *mellonella*. Larvae infected with the wild type or the mutants were incubated at 37°C for 8 days. The survival was reported daily, and no death was registered for the negative control (PBS) and all larvae inoculated with H99, *aap*1Δ and *aap*6Δ died at day 4, suggesting these mutations do not affect virulence. However, larvae inoculated with *aap1*Δ*/aap2*Δ and *aap8*Δ at 37°C died at the fifth day of the experiment, which is statistically significant (p < 0.0001), suggesting these mutations caused virulence attenuation (Fig 3). This result could be due to the combination of low capsule production, in the case of the double mutant, and also the inability of the mutants (*aap8* and *aap1*Δ*/aap2*Δ) to properly uptake amino acids at the high temperature. Therefore, we suggest that these permeases could be important during infection, as it is the case of permeases Aap4 and Aap5 [33].

### The regulation of amino acid uptake

Amino acid uptake is an important feature for virulence as shown by our previous and present work; however, the permeases have large overlapping functions, meaning that they would only be useful as drug target if an inhibitor blocks the transport system by affecting more than one permease in order to attenuate growth, capsule synthesis and ultimately, virulence. The other possibility to use the uptake system as a drug target would be by modulating the global permease gene expression. Our previous work, showed that temperature (37°C), non-preferred carbon source (galactose), nitrogen starvation and the presence of amino acids are environmental signals that induces expression of several permeases [31,33]. However, except for the NCR system [43,75], the genetic elements that control some of these signaling circuits triggered by the nutritional status are not known in *C*. *neoformans*, for example: SPS-sensing and GAAC. Other regulatory systems are well documented in the literature, such as the ones that control high temperature growth, however, it is not known if they affect amino acid uptake and permease expression. Among the mechanisms that control high temperature growth in *C*. *neoformans*, the Ras signaling pathway is very important; therefore, we decided to explore if this pathway has any role in amino acid uptake in this yeast, which is well known to regulate high temperature growth [46,54].

In *S*. *cerevisiae*, the SPS-sensing is involved in a signaling cascade that responds to the amino acid presence leading to permeases expression by Stp1 and Stp2 transcription factors. A homology-based approach identified two Zinc finger transcription factors encoded by genes annotated as CNAG_03366 and CNAG_05392, which have limited but consistent similarity to Stp1 and Stp2 (25% and 34%, respectively). CNAG_03366 (ZNF2) has been enrolled in sexual development and filamentation in *C*. *neoformans* [76,77]. CNAG05392 has been described in a systematic transcription factor profiling [77] and in *C*. *gattii* as a transcription factor involved in zinc homeostasis [78]. In order to address if any of the two genes play a role on amino acid metabolism regulation, we created deletion mutants for both genes (CNU117 and CNU127) as described in S3 Fig. The ability of the mutant strains to grow on individual amino acids as sole nitrogen sources was evaluated, but none of them presented any differences to H99 wild type strain, suggesting these genes may not encode Stp1 and Stp2 homologues.

In order to address the possible cross talk between pathways controlling high temperature growth and amino acid uptake, we analyzed a *C*. *neoformans* serotype A *ras1*Δ::*Neo*^R^ (CBN045), which is largely know as a critical genetic element for high temperature growth [46,49,53]. We reasoned that, if Ras signaling is important for amino acid uptake, a knockout of this gene would affect the growth in amino acid as sole nitrogen source at 30°C and/or 37°C.

This hypothesis was tested and we showed that *ras1* mutant had a significant growth reduction relative to wild type in 11 out of the 15 amino acids tested as nitrogen sources (*p* < 0.05) at 30°C (Table 3). For four amino acids (Ile, Ser, Lys and Trp) growth was not different compared to wild type (Table 3, designated as “NS”) and five amino acids (Ala, Cys, His, Tyr and Thr) could not be tested because they do not support growth (Table 3, designated as “-“). At 37°C, out of 15 amino acids tested, 8 did not promote any growth (designated as “0” in Table 3) and 7 promoted lower growth rates, however, significantly reduced compared to wild type. These results suggest that Ras signaling play a role in amino acid uptake at the nonrestrictive temperature (30°C) and that this feature can also be observed at 37°C. It is noteworthy that this novel phenotype of *RAS*1 is distinct from the phenotypes observed before, which are mostly related to thermo tolerance, due to cytokinesis defects and actin polarization, hyphal differentiation and sexual cycle impairment [47,49]. In this case, the phenotype can be observed at permissive temperature which has no precedent in the literature.

**Table 3:** Percentage of growth in *ras*1Δ (CBN045) strain relative to wild type under the same nutritional condition (single amino acid as sole nitrogen source).

Group	Amino acid	% of growth relative to wild type H99			
		*ras*1Δ at 30°C		*ras*1Δ at 37°C	
Non polar (Hydrophobic)	Ala	-	-	-	-
	Gly	67	***	0	****
	Leu	76	***	49	****
	Ile	108	NS	12	****
	Pro	36	***	47	****
	Val	25	***	16	****
Polar (Uncharged)	Asn	58	****	0	****
	Gln	55	****	0	****
	Ser	89	NS	9	***
	Thr	-	-	-	-
Acid	Asp	58	**	0	****
	Glu	68	****	0	****
Basic	Arg	54	****	0	****
	His	-	-	-	-
	Lys	82	NS	0	****
Sulfur	Met	24	***	33	***
	Cys	-	-	-	-
Aromatic	Phe	76	***	0	****
	Trp	89	NS	18	***
	Tyr	-	-	-	-

(-) indicate the amino acids that were not tested due to low growth; NS means no significant difference compared to wild type; zero means no growth and *p* < 0,03, ** *p <* 0,002, *** p < 0,0002 and **** p < 0,0001.

**Fig 4:**
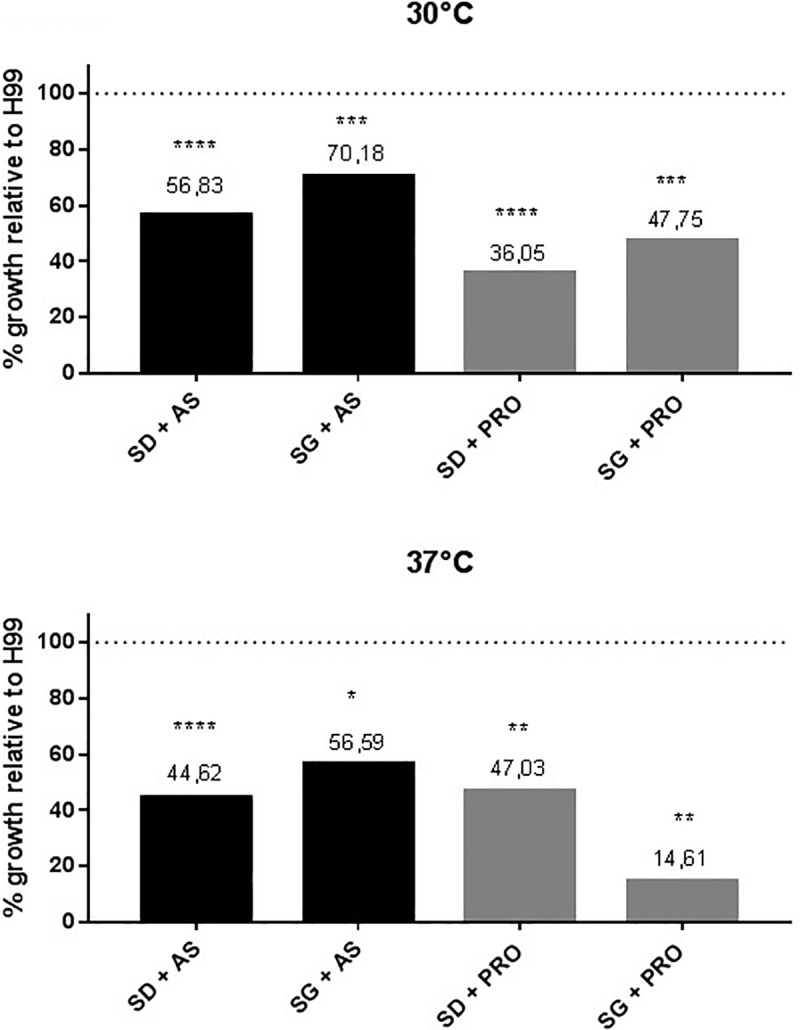
Growth pattern of wild type and *ras1*Δ (CBN045) under non preferred sources of carbon and nitrogen at 30 and 37°C. Growth of the mutant is relative to wild type H99. Controls are the same strains growing in SD (dextrose) or SG (galactose) in ammonium sulfate (AS). Numbers above bars represent the percentage of growth and star the statistical significance of the differences. * *p* < 0,03, ** *p* < 0,002, *** *p* < 0,0002 and **** *p* < 0,0001.

Besides its importance during high temperature growth, amino acid uptake may also be relevant during growth on non-preferred carbon sources, since permease gene expression is elevated in galactose [33]. Since *ras*1Δ is unable to uptake amino acids at 30 and 37°C, we analyzed the growth pattern of *ras*1Δ and wild type on proline (an amino acid that promotes good growth rates) in combination with galactose at both temperatures. Fig 4 shows that growth of *ras1*Δ is reduced compared to wild type in all conditions and temperatures, even though, differences are higher at 37°C. However, at high temperature under non preferred carbon source and proline (SG + Pro), the growth rate of rasΔ is reduced to 14.61% relative to H99. This result suggests that Ras signaling is somewhat required during nutritional (carbon and nitrogen) and heat stress.

### *ras1* mutant fails to induce permease gene expression

**Fig 5:**
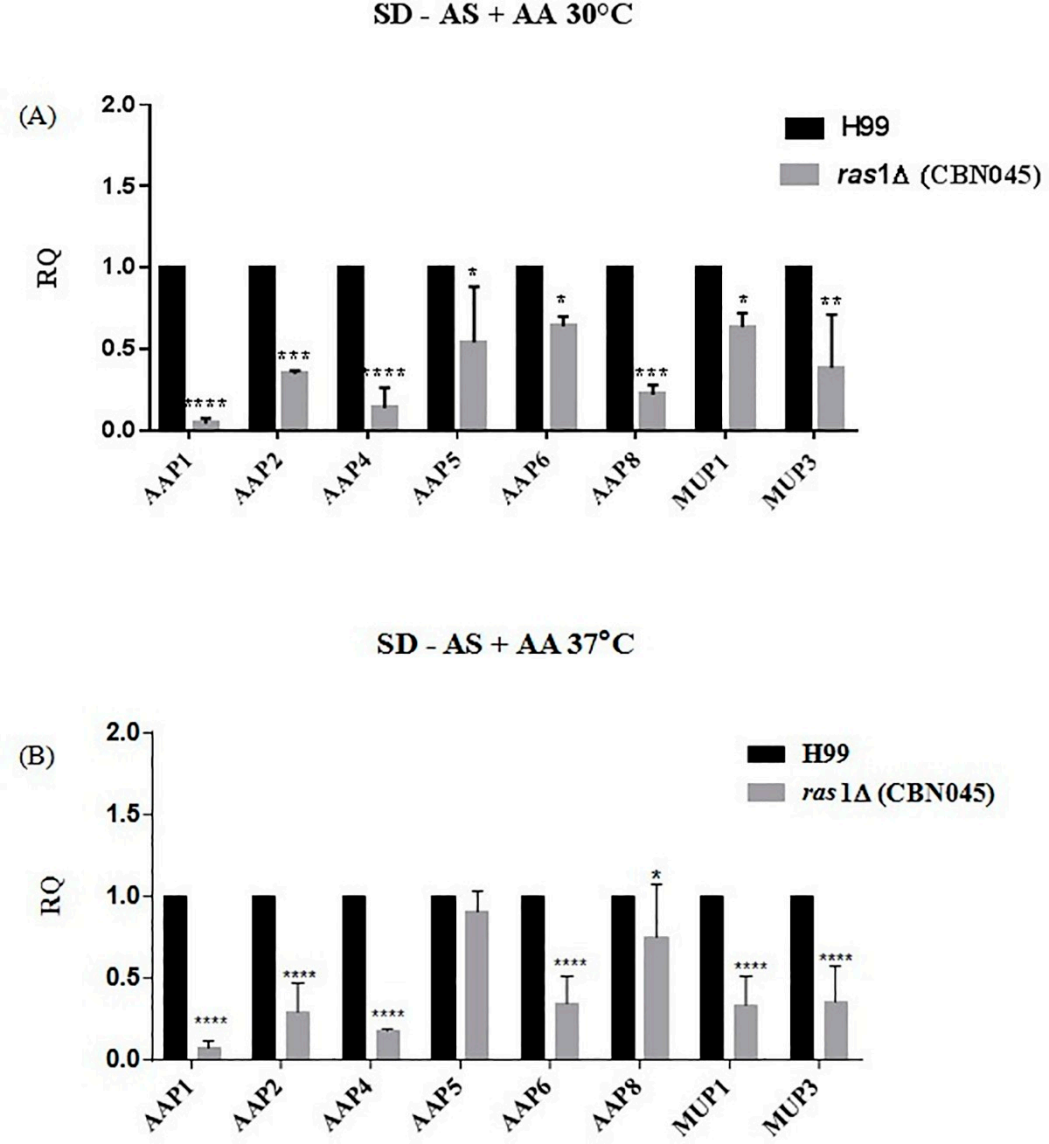
Quantification of permease gene expression on a *ras*1Δ mutant by quantitative real time PCR during growth in (A) YEPD at 30°C and (B) SD added with ammonium sulfate and amino acids (methionine, histidine and tryptophane) at 30°C. *p* < 0,001.

The growth on single amino acid as sole nitrogen source seems to be affected by *RAS*1 deletion at the permissive and also restrictive temperature (30 and 37°C, respectively). Thus, we checked whether the expression of the amino acid permeases would be affected, suggesting a possible mechanism that would explain the growth results. Our hypothesis is that Ras pathway could modulate permease gene expression and therefore, affect amino acid uptake and the growth rate. The expression pattern of eight permease genes was accessed by qPCR in SD without ammonium sulfate (AS) and with amino acids (Trp, His and Met) as nitrogen source, in both temperatures (30 and 37°C). These conditions were selected because permease genes are known to be highly expressed in the presence of amino acids when NCR is relieved. Fig 5 shows that all permeases are strongly repressed in *ras1* mutant compared to wild type at 30°C and 37°C, except for *AAP*5 at higher temperature that has its expression in the mutant at the same levels as in the wild type. These results offer an explanation as to why amino acid uptake in *ras*1Δ is reduced, affecting growth, compared to the wild type strain. Curiously, *AAP*5 has been the most expressed permease in our previous results [31,33]. It is induced over a 100 fold in the presence of amino acids as sole nitrogen source, whereas the others are induced, however, bellow 50 fold. Therefore, it is possible that Aap5 is under a different regulatory mechanism in addition to Ras signaling.

## Discussion

*C*. *neoformans* is an opportunistic pathogen that survives in the human body. This adaptation is due to several virulence factors that favor the pathogen establishment in the host [15]. Adaptation to nutritional challenges may be crucial to survival and virulence [7].

In this manuscript and previous ones, we have evaluated the relevance of amino acid uptake for survival and virulence in opportunistic yeast pathogen. This was done as part of an effort to explore basic biological aspects of an important pathogen as well as to identify putative drug target to develop novel antifungal drugs. Valuable molecular targets are usually specific or structurally very different between the pathogen and the host and often it is desirable to be exposed in the cell surface. *C*. *neoformans* permeases are the main players of amino acid uptake, they are transmembrane proteins located at the cell surface and are significantly different from *H*. *sapiens* APC-like permeases at the primary amino acid sequence and apparently also different at the functional level (Fig 1, Table 1 and S1 Fig).

We carried out a phenotypic analysis of strains containing the individual knockout of eight permeases (*AAP*1, *AAP*2, *AAP*4, *AAP*5, *AAP*6, *AAP*8, *MUP*1 and *MUP*3) and double mutants (*aap*1Δ/*aap*2Δ, *aap*4Δ/*aap*5Δ and *mu*p1Δ/*mup*3Δ). A consolidated picture of these analyses is depicted in Table 4. The compiled data shows that the main phenotypes associated to permease deletion are related to failure to grow on amino acid as sole nitrogen source at higher temperatures, capsule synthesis and ultimately virulence (Table 4), suggesting that amino acid uptake is required under nutritional and temperature stress, a condition often found in the host [15–17].

**Table 4:** Consolidated results of phenotypic analysis of permease mutants. HTG (high temperature growth).

Gene deletion	Relevant phenotypes analyzed for amino acid permease deletion strains						Reference
	HTG	Growth in amino acid at 37°C	Capsule	Stress sensitivity	*G*. *mellonella* model	Mice model	
*aap*1Δ	NA	NA	NA	NA	Virulent	-	This work
*aap*2Δ	NA	NA	NA	NA	Virulent	-	[33]
*aap*1Δ/*aap*2Δ	NA	A	A	NA	Hypovirulent	NT	This work
*aap*3Δ	-	-	-	-	-	-	[35]
*aap*4Δ	NA	NA	NA	NA	Virulent	Virulent	[33]
*aap*5Δ	NA	NA	NA	NA	Virulent	Virulent	[33]
*aap*4Δ/*aap*5Δ	A	A	A	A	Avirulent	Avirulent	[33]
*aap*6Δ	NA	NA	NA	NA	Virulent	-	This work
*aap*7Δ	-	-	-	-	-	-	-
*aap*8Δ	NA	NA	NA	NA	Hypovirulent	-	This work
*mup*1Δ	NA	NA	NA	NA	Virulent	-	[33]
*mup*3Δ	NA	NA	NA	NA	Virulent	-	[33]
*mup*1Δ/*mup*3Δ	NA	A	A	NA	Virulent	-	[33]

NA = not affected; A = affected;— = not tested

Amino acids are probably necessary during stress, since, physiological changes are necessary to adapt the microorganisms to the conditions imposed by the host, which may requires a high demand of protein synthesis. Also, the absence of preferred nitrogen source, which may happen in the host and in the environment, trigger amino acid uptake and that, may serve as a signaling factor indicating nutritional stress.

In *C*. *neoformans* mating is a process that happens in response to nutritional stress and has been linked to amino acids. Specifically, methionine is a ligand of Gpr4 receptor, which triggers capsule biosynthesis and mating. Deletion of this gene leads to the non-production of capsule and filaments during sexual cycle [79]. In *C*. *albicans*, methionine and its high affinity permease, Mup1, have a role on morphogenesis, biofilm formation, survival in the macrophage and virulence [80]. These evidences place amino acid as an important factor, not only as building blocks of proteins, which need to be taken up when preferred nitrogen sources are absent, but also as signaling molecules that trigger various responses to stress.

All the observations regarding amino acid permeases in *C*. *neoformans* that we have collected, such as: quantity and quality of nitrogen and carbon, as well as high temperature modulates the expression of permease genes, lead us to question what genetic elements control amino acid uptake induced by nutritional status. In this paper we rule out two zinc finger transcription factors, which showed sequence homology to *S*. *cerevisiae* Stp1 and Stp2 [42], as responsible for amino acid permease gene activation in response to available amino acids in the environment. Another approach other than homology-based may be necessary to uncover the transcription factors that are at the bottom of a SPS-sensing pathway in order to activate amino acid uptake. Also, a SPS-sensing-like pathway may not exist in *C*. *neoformans* and amino acid uptake maybe controlled in another way. In *C*. *albicans*, an equivalent of *S*. *cerevisiae* Ssy1 exists, but the system works in a different way than the *S*. *cerevisiae* system, regulating all six GAP genes [44,81]

Since amino acid uptake and high temperature growth seem linked, one way to identify genetic elements that regulate permease expression is to analyze the main regulators that control high temperature, since there may be a cross talk between these two processes. Ras pathway is the center of high temperature growth in *C*. *neoformans* [46,52,53]. As we have shown in this paper, it also seems to be at the center of permease expression and amino acid uptake, supporting our cross talk hypothesis. However, Ras1 does not uniformly control the amino acid permease genes, since Aap5 seems to have another level of control, which is in agreement with our previous data, where Aap5 is the strongly induced permease. This result is expected since we know that permease regulation is not a straight simple mechanism; rather it is a complex process involving multiple cues, such as NCR, nitrogen and amino acid availability, which likely trigger multiple pathways promoting differential expression profiles. The same is true for permease expression in *S*. *cerevisiae* and *C*. *albicans* [41,81].

Also, the defective permease expression due to *ras*1Δ mutation is probably the reason why growth on amino acids was affected, reinforcing the arguments that links Ras pathway and amino acid uptake. The observation that *ras1*Δ mutant failed to express some of the permease genes is consistent with the fact that growth is reduced in some amino acids. The use of a secondary carbon source also seems to be important during amino acid uptake at higher temperature, which makes sense, since amino acids as proline, used in this experiment (Fig 4), may also be used as carbon source. It is possible that during heat stress, amino acid serves also as carbon source; therefore, uptake would be important in this circumstance as well.

This is the first time that Ras pathway is connected to nutritional cues in *C*. *neoformans*. In other organisms, for example *S*. *cerevisiae* and *C*. *albicans* this link has been long established [54,82]. Recently authors described sequences at the Ras protein that are conserved in fungi, so that the development of inhibitors that interact with fungal Ras and not to animal Ras may lead to compounds that have high selective toxicity. These differences may be a way to develop novel inhibitors that affect a crucial signaling hub, leading to new treatments for fungal diseases without affecting amino acid uptake by the host [54,83]. This finding is of great relevance and opens the possibility to study the downstream elements of this cascade in *C*. *neoformans* which should of great value for the field.

## Conclusion

Amino acid uptake is an important feature for survival during nutritional (nitrogen and carbon) and heat stress. Failure to assimilate amino acids leads to reduced capsule synthesis and virulence in animal model. In this paper we have found that, besides being important for virulence, amino acid permeases are, at least partially, regulated by the Ras pathway. This is the first report in literature to associate amino acid uptake regulation and a signaling pathway in *C*. *neoformans*.

## Supporting information

S1 Fig Phylogenetic tree showing *C*. *neoformans* and *H*. *sapiens* APC-like permeases. (PPTX)

S2 Fig Restriction map and probe position used in Southern blot for mutant confirmation. (A) *aap1* Δ (B) *aap6*Δ and *aap8*Δ (C) *aap1*Δ*/ aap2*Δ.
(PPTX)

S3 Fig Restriction map and probe position used in Southern blot for mutant confirmation. (A) *stp1*Δ and (B) *stp2*Δ.
(PPTX)

S1 Table Strains used in this work. (DOCX)

S2 Table Primers used in this work. (DOCX)
