# Supplementary figures and images for: Amino acid permeases in Cryptococcus neoformans are required for high temperature growth and virulence; and are regulated by Ras signaling
Source: PLoS One. 2019 Jan 25;14(1):e0211393. doi: 10.1371/journal.pone.0211393 (PMC6347259; doi:10.1371/journal.pone.0211393)

## Slide 1
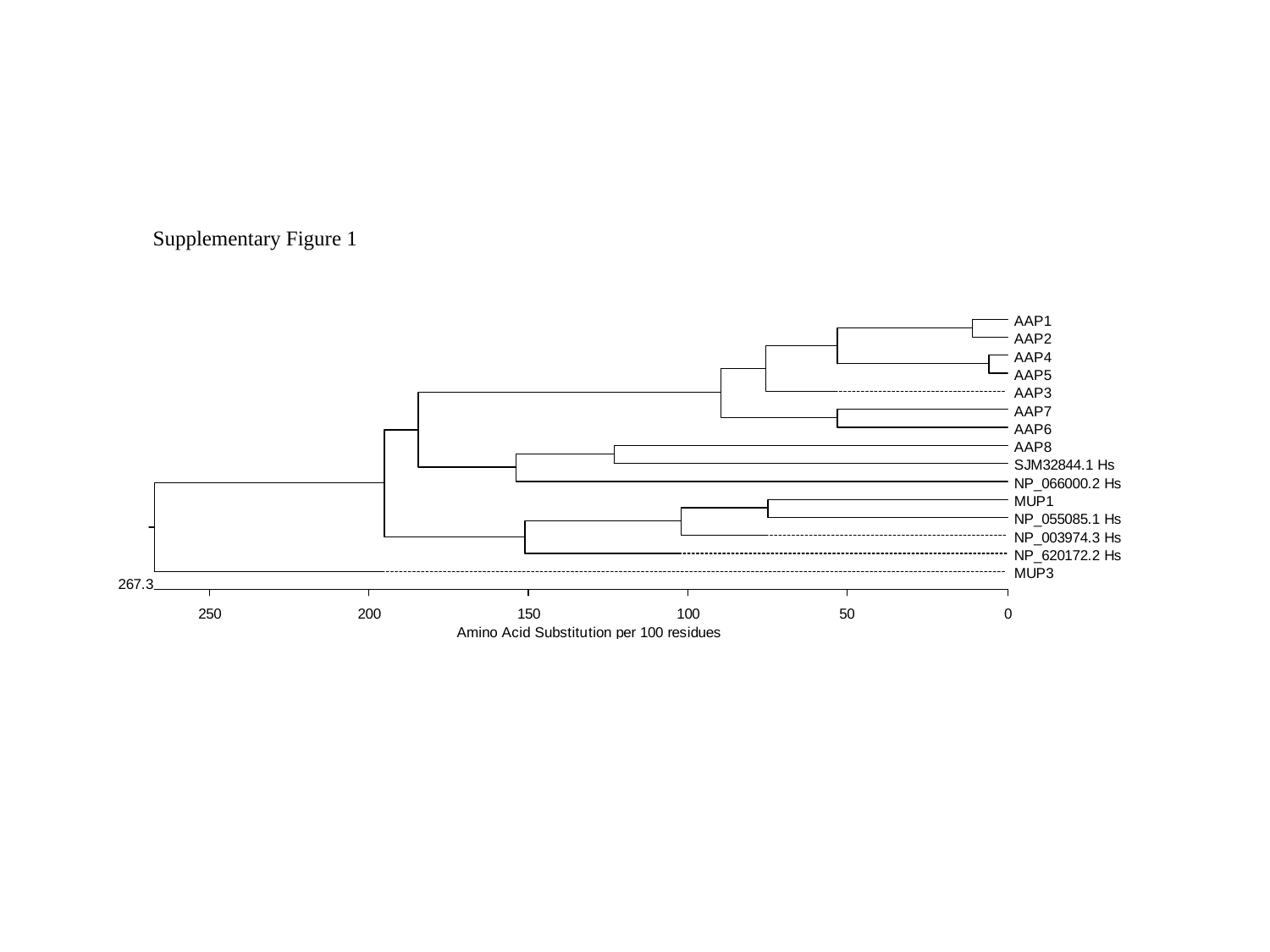

Supplementary Figure 1

Supplement: S1 Fig — (PPTX) [file pone.0211393.s001.pptx]
