# Supplementary material for: Amino acid permeases in Cryptococcus neoformans are required for high temperature growth and virulence; and are regulated by Ras signaling
Source: PLoS One. 2019 Jan 25;14(1):e0211393. doi: 10.1371/journal.pone.0211393 (PMC6347259; doi:10.1371/journal.pone.0211393)
Supplement: S2 Fig — (A) aap1 Δ (B) aap6Δ and aap8Δ (C) aap1Δ/ aap2Δ. (PPTX) [file pone.0211393.s002.pptx]

## Slide 1
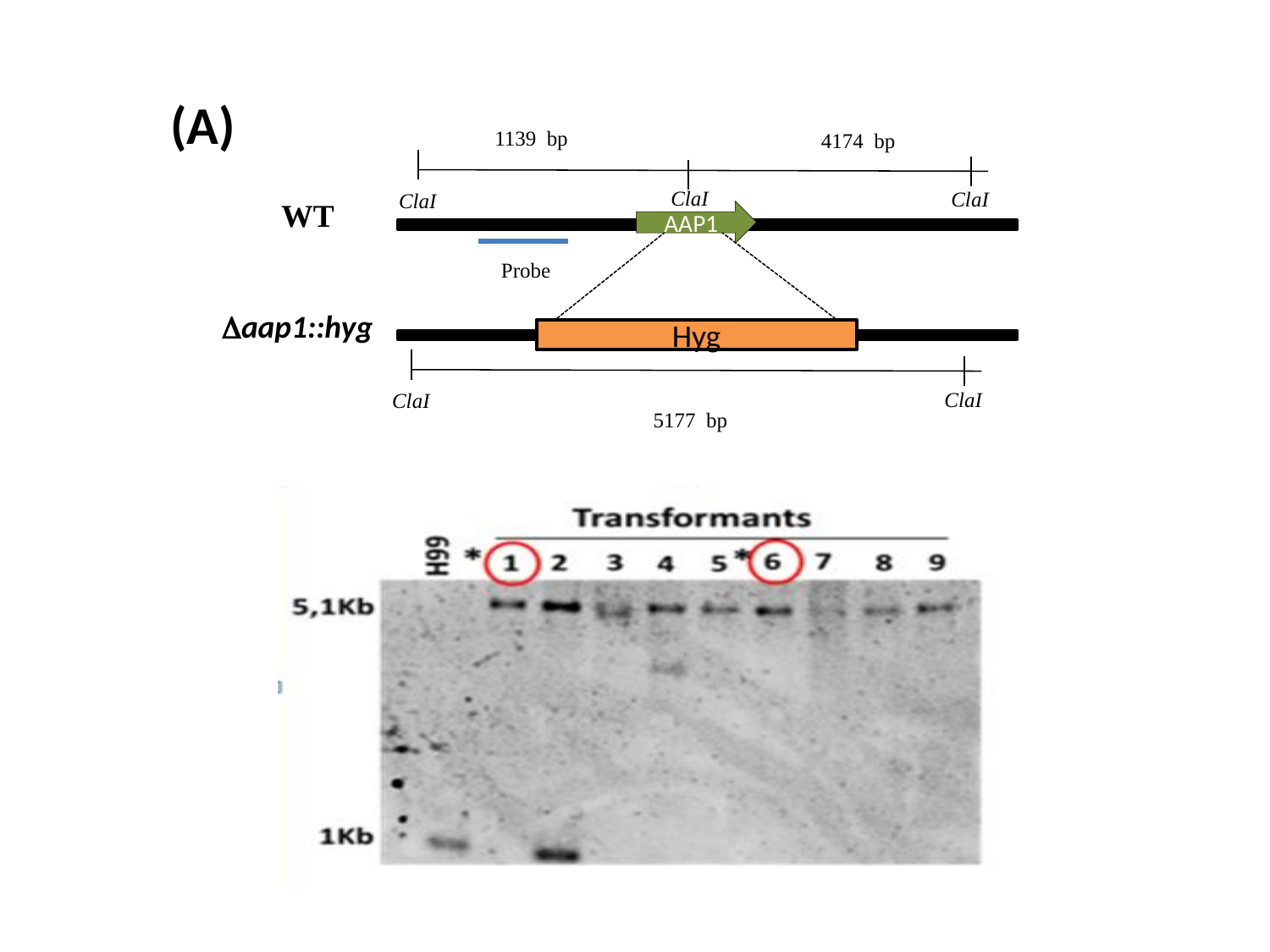

(A)
1139 bp
4174 bp
ClaI
ClaI
ClaI
WT
AAP1
Probe
Δaap1::hyg
Hyg
ClaI
ClaI
5177 bp

## Slide 2
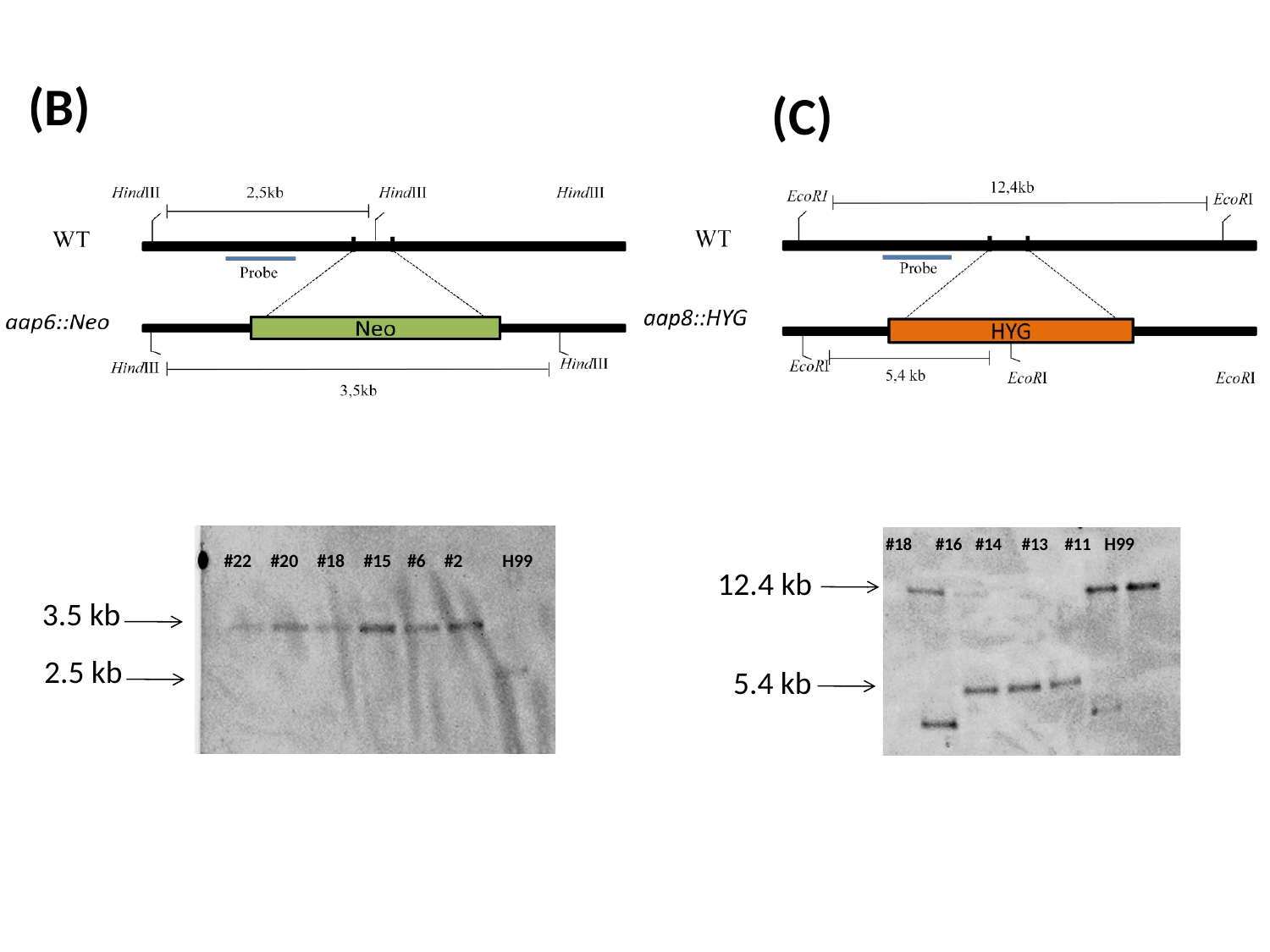

(B)
(C)
#22
#20
#18
#15
#6
#2
H99
3.5 kb
2.5 kb
H99
#18
#16
#14
#13
#11
12.4 kb
5.4 kb

## Slide 3
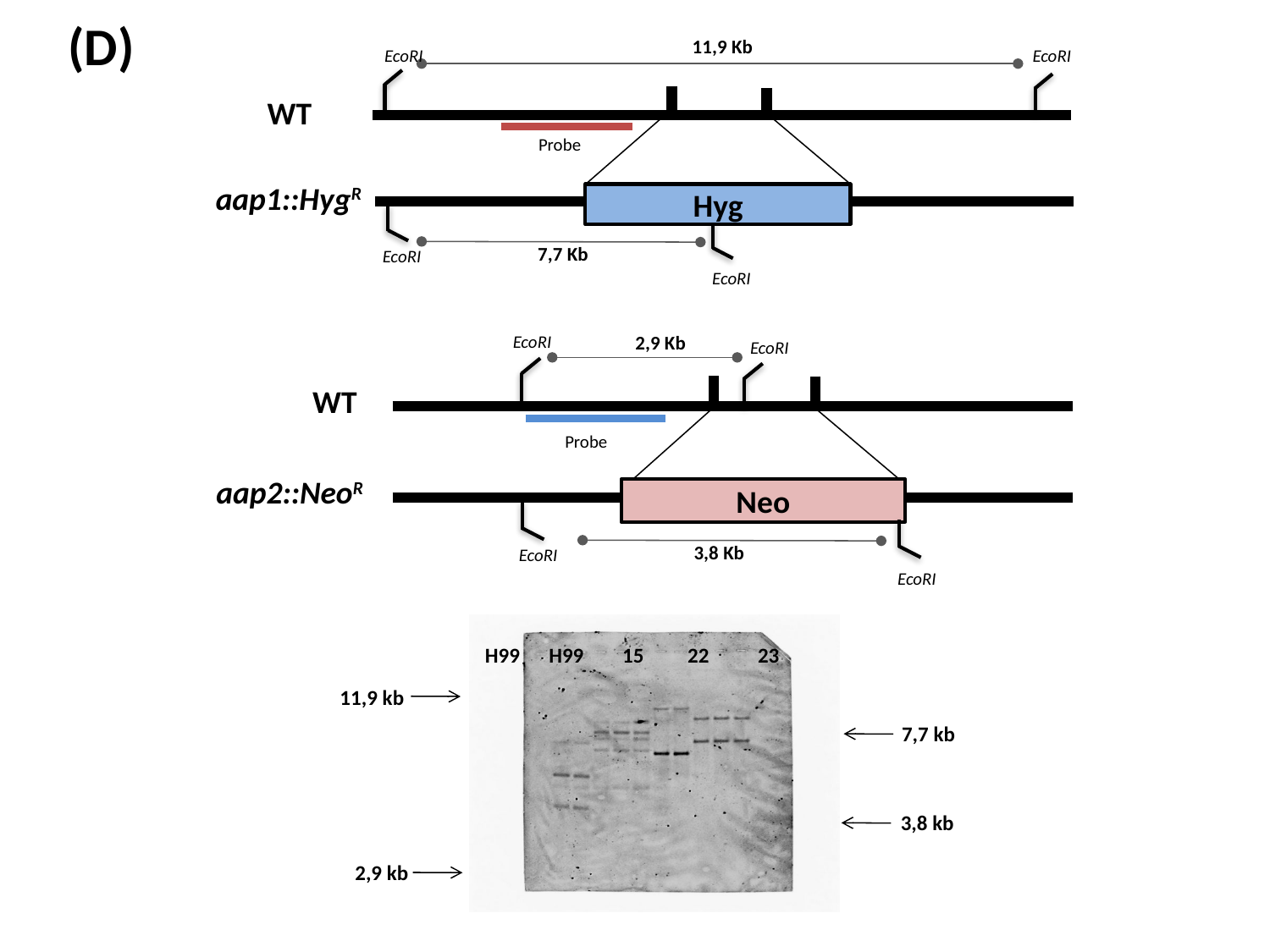

(D)
11,9 Kb
EcoRI
EcoRI
WT
Probe
aap1::HygR
Hyg
7,7 Kb
EcoRI
EcoRI
EcoRI
2,9 Kb
EcoRI
WT
Probe
aap2::NeoR
Neo
3,8 Kb
EcoRI
EcoRI
H99 H99 15 22 23
11,9 kb
7,7 kb
3,8 kb
2,9 kb
