# Supplementary material for: Amino acid permeases in Cryptococcus neoformans are required for high temperature growth and virulence; and are regulated by Ras signaling
Source: PLoS One. 2019 Jan 25;14(1):e0211393. doi: 10.1371/journal.pone.0211393 (PMC6347259; doi:10.1371/journal.pone.0211393)
Supplement: S3 Fig — (A) stp1Δ and (B) stp2Δ. (PPTX) [file pone.0211393.s003.pptx]

## Slide 1
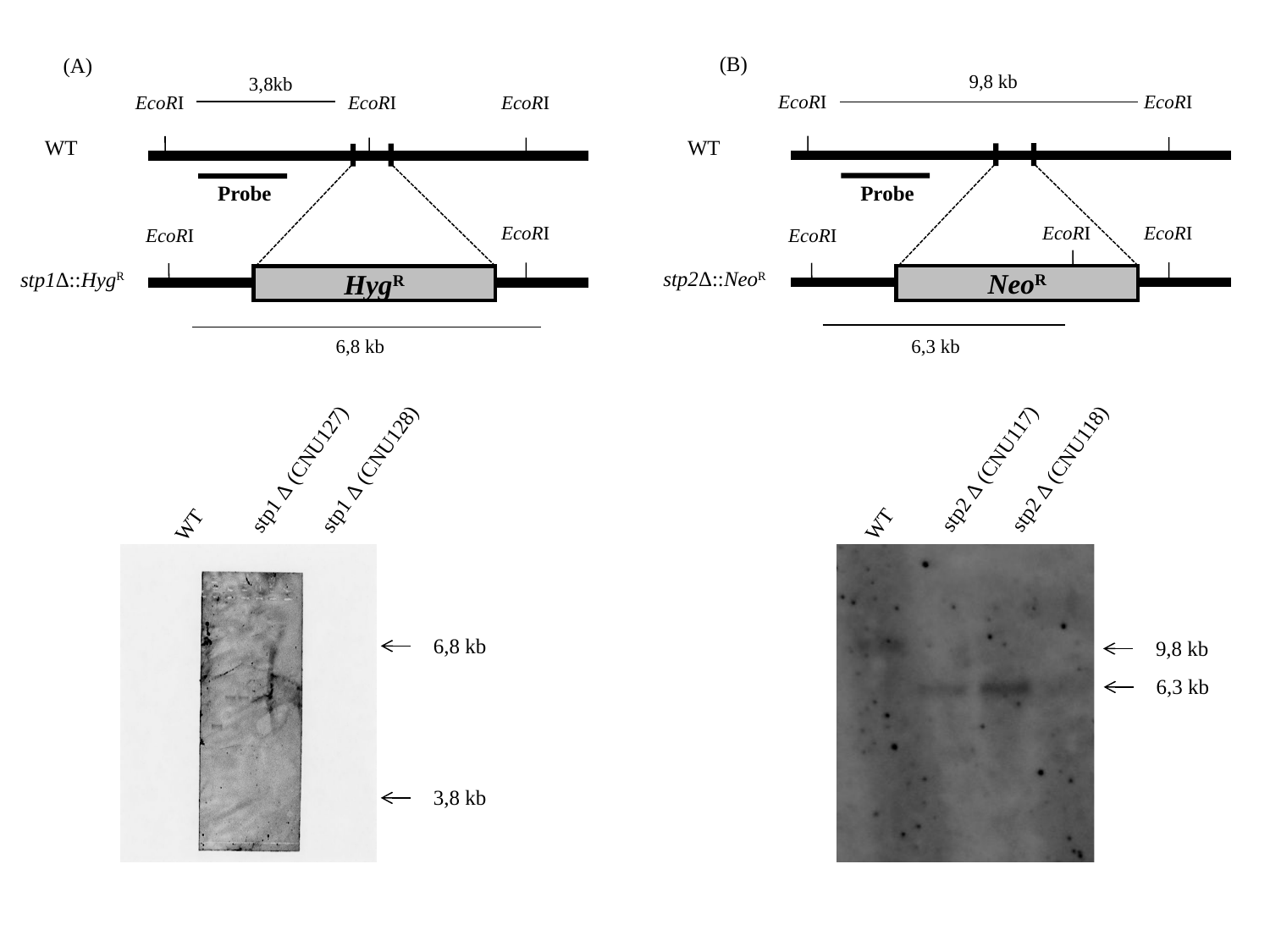

(B)
(A)
9,8 kb
3,8kb
EcoRI
EcoRI
EcoRI
EcoRI
EcoRI
WT
WT
Probe
Probe
EcoRI
EcoRI
EcoRI
EcoRI
EcoRI
stp2Δ::NeoR
stp1Δ::HygR
NeoR
HygR
6,8 kb
6,3 kb
stp2 Δ (CNU117)
stp2 Δ (CNU118)
stp1 Δ (CNU127)
stp1 Δ (CNU128)
WT
WT
6,8 kb
9,8 kb
6,3 kb
3,8 kb
