# Supplementary material for: Amino acid permeases in Cryptococcus neoformans are required for high temperature growth and virulence; and are regulated by Ras signaling
Source: PLoS One. 2019 Jan 25;14(1):e0211393. doi: 10.1371/journal.pone.0211393 (PMC6347259; doi:10.1371/journal.pone.0211393)
Supplement: S1 Table — (DOCX) [file pone.0211393.s004.docx]

S1 Table: Strains used in this work.

| **Strain** | **Strain Background** | **Serotype** | **Genetic markers** | **Origem** |
| --- | --- | --- | --- | --- |
| CNU037 | H99 | A | *aap2Δ::Neo^R^ (#46)* | Martho et al. (2016) |
| CNU093 | H99 | A | *aap6Δ::Neo^R^ (#6)* | This work |
| CNU094 | H99 | A | *aap6Δ::Neo^R^ (#15)* | This work |
| CNU095 | H99 | A | *aap6Δ::Neo^R^ (#18)* | This work |
| CNU096 | H99 | A | *aap6Δ::Neo^R^ (#20)* | This work |
| CNU097 | H99 | A | *aap6Δ::Neo^R^ (#22)* | This work |
| CNU099 | H99 | A | *aap8Δ::Hyg^R^ (#13)* | This work |
| CNU100 | H99 | A | *aap8Δ::Hyg^R^ (#14)* | This work |
| CNU101 | H99 | A | *aap8Δ::Hyg^R^ (#16)* | This work |
| CNU113 | H99 | A | *aap1Δ::Hyg^R^ aap2Δ::Neo^R^ (#21)* | This work |
| CNU114 | H99 | A | *aap1Δ::Hyg^R^ aap2Δ::Neo^R^(#22)* | This work |
| CNU115 | H99 | A | *aap1Δ::Hyg^R^ aap2Δ::Neo^R^* | This work |
| CNU116 | H99 | A | *aap1Δ::Hyg^R^ aap2Δ::Neo^R^* | This work |
| CNU117 | H99 | A | *stp2Δ::Neo^R^ (#2)* | This work |
| CNU118 | H99 | A | *stp2Δ::Neo^R^ (#3)* | This work |
| CNU127 | H99 | A | *stp1Δ:Hyg^R^ (#1)* | This work |
| CNU128 | H99 | A | *stp1Δ:Hyg^R^ (#14)* | This work |
| CBN045 | H99 | A | *ras*1Δ::*Neo^R^* | Alspaugh et al., 2000 |
| H99 | - | A | Wild type | Our collection |
