# Supplementary material for: Amino acid permeases in Cryptococcus neoformans are required for high temperature growth and virulence; and are regulated by Ras signaling
Source: PLoS One. 2019 Jan 25;14(1):e0211393. doi: 10.1371/journal.pone.0211393 (PMC6347259; doi:10.1371/journal.pone.0211393)
Supplement: S2 Table — (DOCX) [file pone.0211393.s005.docx]

S2 Table: Primers used in this work.

| **Code** | **Sequence (5´to 3´)** | **F/R** | **Use** |
| --- | --- | --- | --- |
| PRCP185 | GCCTTATGGTATACTCTATGATG | F | CNAG_02539.2 qPCR AAP1 |
| PRCP186 | CCGTATGCCCGAGCACCGAGG | R | CNAG_02539.2 qPCR AAP1 |
| PRCP188 | TCTGGCTCCCCAGAAGTTAATG | R | CNAG_07902.2 qPCR AAP2 |
| PRCP191 | CGAGGCAAAGAACCCACG | F | CNAG_00597.2 qPCR AAP4 |
| PRCP192 | AATCAAGATGCAAGCGTTTATG | R | CNAG_00597.2 qPCR AAP4 |
| PRCP193 | ACTTACTTGGACCTCTATCCTC | F | CNAG_07367.2 qPCR AAP5 |
| PRCP194 | TTTTCGGATCAGCTTGAAACC | R | CNAG_07367.2 qPCR AAP5 |
| PRCP195 | CCTTGAAAGACCGTTTCGGC | F | CNAG_07449.2 qPCR AAP6 |
| PRCP196 | TGTCACAAGTGTTGGGTCATTG | R | CNAG_07449.2 qPCR AAP6 |
| PRCP199 | TCTCTTTCTAGGGATTCTTATC | F | CNAG_00574.2 qPCR AAP8 |
| PRCP200 | CTCCGCCATATGGGCAGAAGC | R | CNAG_00574.2 qPCR AAP8 |
| PRCP284 | GTTCAACTTCATTGTTGCCC | F | Mup1 qPCR |
| PRCP285 | AGGAACACGATCCCTAGTC | R | Mup1 qPCR |
| PRCP286 | TATGTGGCAGGCGAAATGC | F | Mup3 qPCR |
| PRCP287 | CCTTGCAGTTGTGTACAATC | R | Mup3 qPCR |
| PRCP228 | ATATGTACAATTAAACAGCAA | F | AAP2 deletion construct |
| PRCP229 | CTCCAGCTCACATCCTCGCAGTCCTTCTAGAGGAAGTGAATGAATTG | R | AAP2 deletion construct |
| PRCP230 | CAATTCATTCACTTCCTCTAGAAGGACTGCGAGGATGTGAGCTGGAG | F | AAP2 deletion construct |
| PRCP231 | CAACTAACTATGATTTCTAGAAGAGATGTAGAAACTAGCTTCC | R | AAP2 deletion construct |
| PRCP232 | GGAAGCTAGTTTCTACATCTCTTCTAGAAATCATAGTTAGTTG | F | AAP2 deletion construct |
| PRCP233 | GATGCCGGTACCTTTGATGGG | R | AAP2 deletion construct |
| PRCP315 | GATGGAGGAATCCAGATACG | F | AAP6 deletion construct |
| PRCP316 | CATACATAACAGCTGAGAGAAGTGCGAGGATGTGAGCTGGAG | F | AAP6 deletion construct |
| PRCP317 | CTCCAGCTCACATCCTCGCACTTCTCTCAGCTGTTATGTATG | R | AAP6 deletion construct |
| PRCP318 | GGAAGCTAGTTTCTACATCTCTTCTGAGATTGAAATACGGGCGGTGG | F | AAP6 deletion construct |
| PRCP319 | CCACCGCCCGTATTTCAATCTCAGAAGAGATGTAGAAACTAGCTTCC | R | AAP6 deletion construct |
| PRCP320 | CGTCGATAGTTAGATAAAGC | F | AAP6 deletion construct |
| PRCP321 | GTACGTGCTCTCCAGTTGGG | R | AAP6 deletion construct |
| PRCP322 | TCGTCGTTGTTCTTATCGG | F | AAP8 deletion construct |
| PRCP323 | TCGTCGTTGTTCTTATCGG | F | AAP8 deletion construct |
| PRCP324 | CCCAATTTTTTACCATCTTGGCCTGCGAGGATGTGAGCTGGAG | R | AAP8 deletion construct |
| PRCP325 | CTCCAGCTCACATCCTCGCAGGCCAAGATGGTAAAAAATTGGG | F | AAP8 deletion construct |
| PRCP326 | GGAAGCTAGTTTCTACATCTCTTCAAGTCAAGTGGATGTTCGAATC | R | AAP8 deletion construct |
| PRCP327 | GATTCGAACATCCACTTGACTTGAAGAGATGTAGAAACTAGCTTCC | F | AAP8 deletion construct |
| PRCP328 | TCCGGTGGTGCATAGTCCAG | R | AAP8 deletion construct |
| PRCP329 | GCAGTTCTCACCATCAGGTG | F | AAP1 deletion construct |
| PRCP330 | CTCCAGCTCACATCCTCGCACTTTGCTGTTATGCGCGCTT | R | AAP1 deletion construct |
| PRCP331 | AAGCGCGCATAACAGCAAAGTGCGAGGATGTGAGCTGGAG | F | AAP1 deletion construct |
| PRCP332 | CAAAGCTTCATCAAACCACCGAAGAGATGTAGAAACTAGCTTCC | R | AAP1 deletion construct |
| PRCP333 | GGAAGCTAGTTTCTACATCTCTTCGGTGGTTTGATGAAGCTTTG | F | AAP1 deletion construct |
| PRCP334 | GGGTTGAACCTGTACTTTTTG | R | AAP1 deletion construct |
| PRCP335 | CGCCGATTCTGCGAGATCACCCATGGAGGCCGAATTCATGTTCCCCGACCTTCCCG | F | AAP1 deletion construct |
| PRCP353 | CCTTGCAGAAGGTTTTGGATGA | F | STP1 deletion construct |
| PRCP354 | CTCCAGCTCACATCCTCGCAGTTGTAAACCAGCTTTTTAAGTAG | R | STP1 deletion construct |
| PRCP355 | CTACTTAAAAAGCTGGTTTACAACTGCGAGGATGTGAGCTGGAG | F | STP1 deletion construct |
| PRCP356 | TACTTGCAGCGAACAAAAGTCTTGAAGAGATGTAGAAACTAGCTTCC | R | STP1 deletion construct |
| PRCP357 | GGAAGCTAGTTTCTACATCTCTTCAAGACTTTTGTTCGCTGCAAGTA | F | STP1 deletion construct |
| PRCP358 | CGTCTACTTCGGTTCCCGTC | R | STP1 deletion construct |
| PRCP359 | AGCCGATATACGACAGCCGC | F | STP1 deletion construct |
| PRCP360 | CGCTCGGGCATCTGTCCAAC | R | STP1 deletion construct |
| PRCP361 | CCGTCCTTCCATTCCCCAACG | F | STP2 deletion construct |
| PRCP362 | CCAGCTCACATCCTCGCATTCGATTTCGATTTTGAGCTAAAG | R | STP2 deletion construct |
| PRCP363 | CTTTAGCTCAAAATCGAAATCGAATGCGAGGATGTGAGCTGGAG | F | STP2 deletion construct |
| PRCP364 | TCGATAACTCGATTTAGATTAACGGAAGAGATGTAGAAACTAGCTTCC | R | STP2 deletion construct |
| PRCP365 | GGAAGCTAGTTTCTACATCTCTTCCGTTAATCTAAATCGAGTTATCGA | F | STP2 deletion construct |
| PRCP366 | CACCAACAGGAGAGAAAAGGG | R | STP2 deletion construct |
| PRCP367 | CGGAGAGATCAGCCGTGTTAC | F | STP2 deletion construct |
| PRCP368 | GAAGAGATGGCTGCGGATGG | R | STP2 deletion construct |
